# MERITXELL: The Multifrequency Experimental Radiometer with Interference Tracking for Experiments over Land and Littoral—Instrument Description, Calibration and Performance

**DOI:** 10.3390/s17051081

**Published:** 2017-05-10

**Authors:** Jorge Querol, José Miguel Tarongí, Giuseppe Forte, José Javier Gómez, Adriano Camps

**Affiliations:** Remote Sensing Lab (RSLab), Department of Signal Theory and Communications (TSC), Universitat Politècnica de Catalunya—BarcelonaTech (UPC), Jordi Girona 1-3, Campus Nord, D4 Building, 08034 Barcelona, Spain; jose_miguel_tarongi@tsc.upc.edu (J.M.T.); giuseppe.forte@tsc.upc.edu (G.F.); josegomez931994@hotmail.com (J.J.G.)

**Keywords:** microwave radiometry, multiband radiometer, data fusion, radio-frequency interference

## Abstract

MERITXELL is a ground-based multisensor instrument that includes a multiband dual-polarization radiometer, a GNSS reflectometer, and several optical sensors. Its main goals are twofold: to test data fusion techniques, and to develop Radio-Frequency Interference (RFI) detection, localization and mitigation techniques. The former is necessary to retrieve complementary data useful to develop geophysical models with improved accuracy, whereas the latter aims at solving one of the most important problems of microwave radiometry. This paper describes the hardware design, the instrument control architecture, the calibration of the radiometer, and several captures of RFI signals taken with MERITXELL in urban environment. The multiband radiometer has a dual linear polarization total-power radiometer topology, and it covers the L-, S-, C-, X-, K-, Ka-, and W-band. Its back-end stage is based on a spectrum analyzer structure which allows to perform real-time signal processing, while the rest of the sensors are controlled by a host computer where the off-line processing takes place. The calibration of the radiometer is performed using the hot-cold load procedure, together with the tipping curves technique in the case of the five upper frequency bands. Finally, some captures of RFI signals are shown for most of the radiometric bands under analysis, which evidence the problem of RFI in microwave radiometry, and the limitations they impose in external calibration.

## 1. Introduction

### 1.1. Multiband Microwave Radiometers

Microwave Radiometry (MWR) is a passive remote sensing technique that consists of measuring the spontaneous emission of electromagnetic energy radiated by all bodies at a physical temperature higher than 0 K. Nowadays, MWR has become a common and powerful tool for Earth remote sensing because of its high accuracy and large swath, despite its low spatial resolution. MWR allows measuring remotely atmospheric and geophysical parameters with the study and analysis of the received electromagnetic spontaneous emission. Among them, prominent examples are soil moisture, sea ice concentration, snow coating, rain rate over soil and ocean, sea surface salinity and temperature, wind speed over the sea, sea oil spills tracking, atmospheric temperature profiles, water vapor profiles, or cloud liquid water content [1]. However, each one of these parameters can be only measured at some particular frequency bands for several reasons such as penetration depth, resonance frequencies of the molecules or transmittance of the atmosphere, etc. The most frequently used bands for some of the already mentioned applications are listed in Table 1.

Almost all geophysical parameters can be measured at multiple microwave frequency bands. Therefore, multiband microwave radiometers are designed to combine data retrieved from several bands in order to achieve improved measurement. Four examples of multiband microwave radiometers, two airborne and two spaceborne, are mentioned subsequently:

- The Helsinki University of Technology RADiometer (HUTRAD) for remote sensing is an airborne radiometer which includes a non-imaging subsystem that operates at six frequencies between 6.8 and 94 GHz, with vertically and horizontally polarized channels at each frequency [3].
- The Polarimetric Scanning Radiometer (PSR) is an airborne instrument which operates at 10.7, 18.7, 37, and 89 GHz, and measures the first three modified Stokes’ parameters. It has two-axis scanning capability and provides polarimetric data for microwave emission studies of both ocean and land surfaces, as well as atmospheric clouds and precipitation [4].
- The Special Sensor Microwave Imager/Sounder (SSMI/S) is a spaceborne mission which includes a 24-channel single conically scanning radiometer, and represents the most complex operational satellite passive microwave imager/sounding sensor ever flown with capabilities to profile the mesosphere. The receiver subsystem accepts the energy from the six antenna feeds and provides amplification and filtering to the 24 output channel located at the 19, 22, 37, 50–60, 91, 150, and 183 GHz frequency bands [5].
- The Advanced Microwave Scanning Radiometer for the Earth Observing System (AMSR-E) is a six-frequency dual-polarized total-power passive microwave spaceborne radiometer that observes water-related geophysical parameters supporting global change science and monitoring efforts. The supported frequency bands include 6.925, 10.65, 18.7, 23.8, 36.5, and 89.0 GHz [6].

In addition to MWR, other sensors working at different bands (e.g., radar and optical sensors) can retrieve complementary data useful to develop geophysical models with improved accuracy. In the field of remote sensing, the combination of multi-sensor measurements is better known as data fusion.

One example of data fusion is the combination of Global Navigation Satellite Systems-Reflectometry (GNSS-R), and L-band MWR. GNSS-R is an emerging technique based on the acquisition of the forward scattered GNSS signals over the Earth’s surface [7,8]. GNSS-R performs worse than L-band MWR in terms of accuracy due to its higher sensitivity to surface roughness (i.e., speckle noise), but it has a much better spatial resolution, specially when the coherent reflection process dominates over the incoherent one [8]. Therefore, both techniques can be used together to improve the spatial resolution of the overall measurement since they are sensitive to the same geophysical parameters, for instance in the case of soil moisture [9]. The combination of MWR and GNSS-R data has also been used to improve sea surface salinity retrievals, by correcting the sea state impact on the brightness temperature using GNSS-R observables [10].

Another example of data fusion with a similar approach is the combination of MWR measurements with optical sensor data. In [11] a downscaling approach to improve the spatial resolution of soil moisture estimates obtained by the SMOS (Soil Moisture and Ocean Salinity) mission with the use of higher resolution visible/infrared (VIS/IR) satellite data is presented. The results of this study show a strong correlation between VIS/IR satellite data and soil moisture status.

### 1.2. The Problem of Radio-Frequency Interference

Microwave radiometers are among the most sensitive instruments, as they are designed to fulfill stringent requirements. This implies that any unexpected signal, apart from the radiometric noise, present at the same radiometric band under analysis, causes a bias on the final power measurement.

Passive radiometric bands are usually protected by the international regulations, which forbid any electromagnetic emission in these bands. However, MWR suffers from Radio-Frequency Interference (RFI) in many bands, boosted by the pervasive use of wireless communications. RFI signals are those that are intentionally or unintentionally emitted in protected frequency bands, which degrade the performance of the receivers. There are a huge number documented cases of RFI in MWR applications. Two examples of them are the case of SMOS described in [12], and the case of AMSR-E described in [13].

In the recent years, many studies have been done in order to solve the problem of RFI in MWR. Algorithms for RFI localization are being developed to find and switch the RFI sources off (e.g., [14]). Another approach to solve the RFI problem is the development of RFI detection and mitigation techniques, in order to deal with the RFI problem from the receiver side. There are many different approaches for RFI detection and mitigation that have been developed for microwave radiometry such as time-frequency blanking [15,16], normality tests [17,18] or polarimetric analysis [19,20] among others.

### 1.3. MERITXELL Instrument

MERITXELL is a ground-based multisensor instrument that includes a multiband dual-polarization microwave radiometer, a GNSS reflectometer, and several optical sensors. Its main goals are to test data fusion techniques, and to implement different kinds of RFI detection/mitigation techniques.

MERITXELL has been developed at the Remote Sensing Laboratory (RSLab) at Polytechnic University of Catalonia-BarcelonaTech (UPC). Three Ph.D theses, and a number of graduate and undergraduate students, have been working in the development of this instrument since 2007. The first Ph.D thesis [21] was devoted to the design and development of the radio-frequency systems. The second one [22] was devoted to the system integration, and temperature stabilization. The third one was devoted to the calibration, characterization, and control of the instrument. The purpose of this paper is to describe the instrument MERITXELL, as well as to describe the calibration process, and some measurements.

This paper is divided into six sections as follows. Section 2 details the design and implementation of MERITXELL and its different sub-systems. Section 3 describes the control and measurement system of the multiband microwave radiometer. Section 4 explains the calibration and characterization procedure. Section 5 presents several examples of RFI signals captured with the instrument. Finally, Section 6 summarizes the main conclusions and remarks of this work.

## 2. Instrument Design

MERITXELL is a ground-based multisensor instrument which includes a multiband dual-polarization microwave radiometer with several additional features that make it unique as compared to other instruments. Its name stands for Multifrequency Experimental Radiometer with Interference Tracking for eXpEriments over Land and Littoral. MERITXELL has been designed to fulfill the following requirements:

- to include all passive radiometric bands up to 100 GHz used for Earth observation from a satellite (this excludes the 50–60 GHz bands ([1], Chapter 1)).
- to be built using a flexible back-end system to allow custom signal processing techniques for RFI detection, localization and mitigation, and data fusion algorithms.
- to include several optical sensors such as a thermal infrared and a multispectral camera with visible and near-infrared bands. These optical sensors are able to retrieve the complementary measurements used by data fusion algorithms.
- to include a GNSS reflectometer to retrieve GNSS-R data for data fusion algorithms.
- to be mounted in a ground-based mobile platform that allows to transport it, and to point it to any desired position in azimuth and elevation.

**Figure 1:**
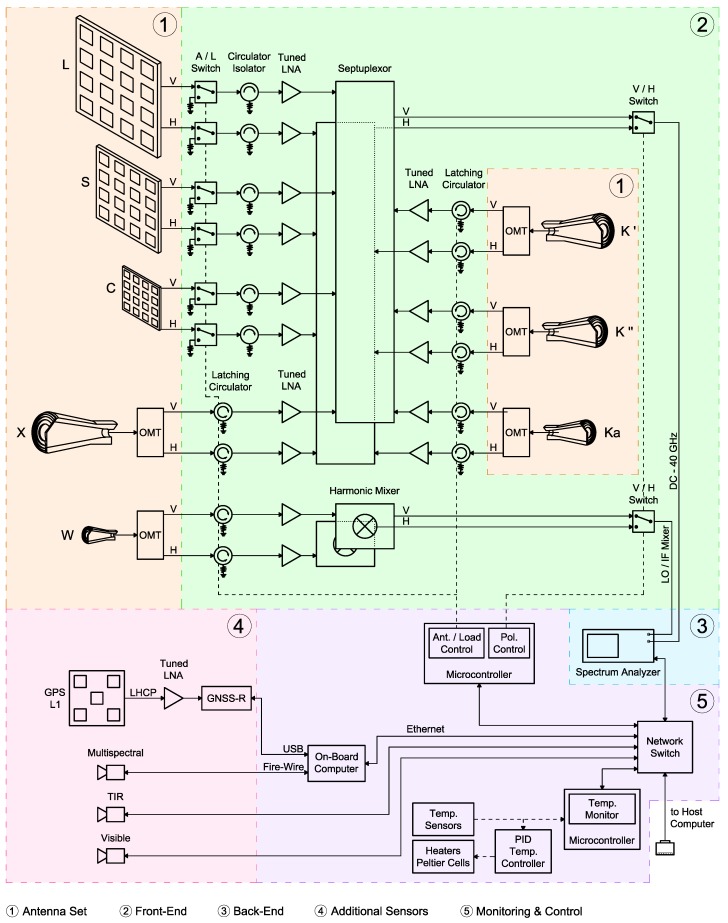
MERITXELL overall block diagram. The design has been divided in five sub-systems: (**1**) antenna set; (**2**) front-end; (**3**) back-end; (**4**) additional sensors; and (**5**) monitoring & control.

An overall diagram of all constituent blocks of MERITXELL is shown in Figure 1. Preliminary versions of the instrument and its sub-systems were already introduced in [21,22,23,24]. Nevertheless, the aim of this section is to present the final version of each sub-system, as well as the final integrated instrument.

The following subsections describe each one of the different parts of the instrument, including the multiband radiometer, the temperature stabilization system, the additional sensors, and the enclosure and mobile unit.

### 2.1. Radiometer Assembly

The main part of MERITXELL is the eight-band dual-polarization total-power microwave radiometer, designed with a flexible back-end structure ready to implement RFI detection, localization, and mitigation algorithms. The description of the radiometer assembly is divided in three sub-sections: antenna set, front-end, and back-end.

#### 2.1.1. Antenna Set

**Figure 2:**
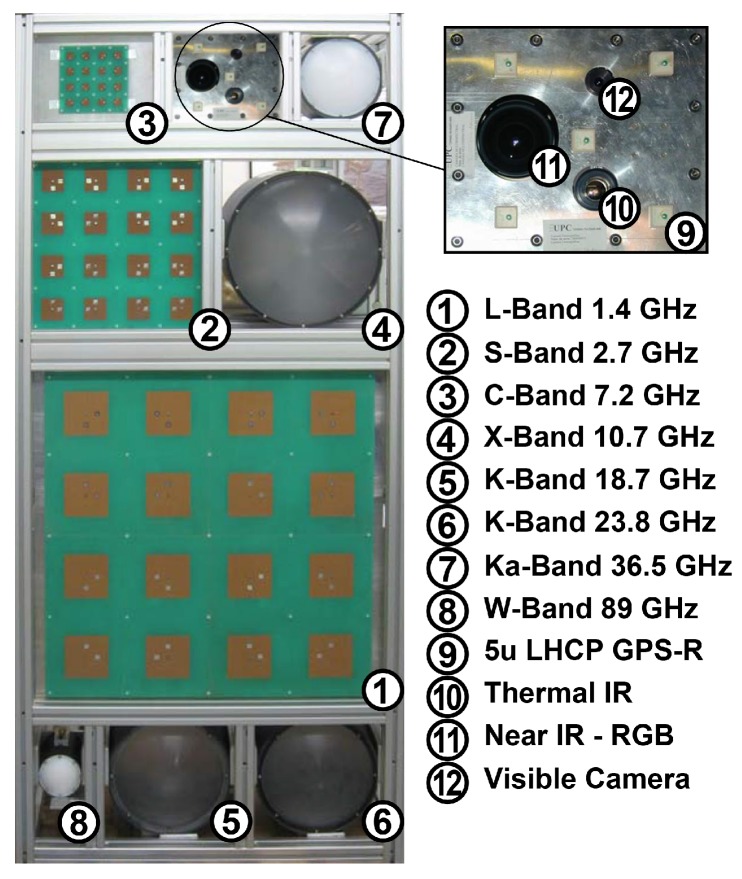
Front view of the antenna set and additional sensors of MERITXELL.

The antenna set of MERITXELL is formed by eight dual linear polarization antennas that collect the electromagnetic radiation in different passive Earth observation bands, commonly used in microwave radiometry. These eight bands are located into seven bands named: L-band (1.400–1.427 GHz), S-band (2.69–2.70 GHz), C-band (7.14–7.23 GHz), X-band (10.6–10.7 GHz), K-band (18.6–18.8 GHz, and 23.6–24.0 GHz), Ka-band (36–37 GHz), and W-band (86–92 GHz). For the sake of clarity, each of the bands at K-band is coined with a different name in this work. The 18.6–18.8 GHz band is named K’, whereas the 23.6–24.0 GHz band is named K”. Figure 2 shows a front view of the eight antennas of instrument MERITXELL without their radome.

In the case of the L-, S-, and C-band, the antennas for each band are 4 × 4 patch arrays with a 25${}^{\circ }$ beamwidth at −3 dB, a Main Beam Efficiency (MBE) of 95%, a Cross-Polarization Isolation (CPI) better than 35 dB in the main beam (i.e., −3 dB beamwidth), and a radiation efficiency $\eta_{A}$ of 95%. Parameters obtained for the antennas have been measured at UPC anechoic chamber as mounted in the whole system. Each one of the patches that conform these array antennas is a dual-polarization coaxial-fed microstrip antenna printed in a 0.6 mm fiberglass circuit board with dielectric air. The signals collected by the 16 patches are combined with different weights depending on the position in the array by means of a microstrip power combiner circuit for each polarization, vertical (V) and horizontal (H). Barlett (triangular) tapering is applied in both directions to reduce the side lobes and improve the main beam efficiency, a critical parameter for microwave radiometer antennas. Eventually, the signal output of each power combiner is guided through a coaxial cable to the front-end stage. These antennas have been developed following the novel architecture implemented in the L-band AUtomatic RAdiometer (LAURA), described in [25], because of their excellent performance. In fact, the one used for the L-band measurements is an improved replica of the LAURA antenna, whereas the S-, and C-band antennas are scaled versions of the previous one.

**Figure 3:**
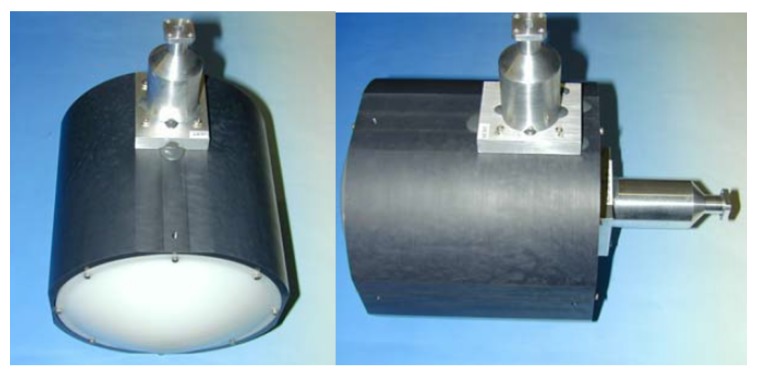
Corrugated horn with its Fresnel lens used in the Ka-band with the corresponding output waveguides for V and H polarizations.

For the X-, K-, Ka-, and W-band, the chosen antenna for each one is a corrugated horn combined with a Fresnel lens that produces a Gaussian beam. The corrugated horns contribute to increase the bandwidth, as well as the Main MBE and CPI figures as compared to other kinds of horn antennas. The resulting antennas have a beamwidth at −3 dB with a maximum of 6${}^{\circ }$ at X-band, and a minimum of 3.2${}^{\circ }$ at W-band, their MBE is about 98%, their CPI is higher than 37 dB at the main beam (i.e., −3 dB beamwidth), and the radiation efficiency $\eta_{A}$ is about 99%. The signal collected by each horn is separated into V and H polarization signals using an Orthomode Transducer (OMT). Eventually, each one of the OMT outputs feeds its corresponding waveguide to the front-end stage. Figure 3 shows the antenna used for the Ka-band with the corresponding output waveguides for V and H polarizations. Eventually, Table 2 summarizes the features of the antenna set built in MERITXELL.

#### 2.1.2. Front-End Stage

The front-end stage is composed by several sub-stages whose purpose is to condition the signals collected by the antenna set, and then, to guide them to the back-end stage. These sub-stages are depicted in the overall diagram in Figure 1. The first sub-stage is used to calibrate the drifts of the amplifiers with the help of matched loads with known physical temperature. For the L-, S-, and C-band, the calibration stage is composed by a Single-Pole Dual-Through (SPDT) switch, that commutes between the signal coming from the antenna, and the matched load, followed by a circulator that acts as an isolator. For the X-, K-, Ka-, and W-band, the scheme is similar but simplified due to the existence of the latching circulators. There is one matched load per band, and polarization, and their physical temperature is measured by means of a digital temperature sensor attached to each one of them.

After that, the signal power is boosted with tuned amplifiers, one for each band and polarization. All these tuned amplifiers have a gain of at least 60 dB [22] in the band-pass frequency, with gain flatness ≤ 1.5 dB, noise figure ≤ 2.4 dB at the highest frequency band, and 1 dB compression point ≥ 5 dBm for the worst case.

Then, the output of each amplifier of the seven lower frequency bands (L-, S-, C-, X-, K-, and Ka-band) are combined using two different septuplexors (7-to-1 tuned power combiners), one for the vertical and another for the horizontal polarization. After that, a SPDT switch commutes between the output of the two septuplexors, and finally, the combined signal reaches the back-end stage.

Furthermore, the W-band signal is treated in a different way as compared to the signal of the lower bands after the amplification stage. Since the bandwidth of the back-end stage can reach up to 40 GHz, the W-band signal is first down-converted to an Intermediate Frequency (IF) much lower than 40 GHz. This process is done using two specific harmonic mixers, one for the vertical, and another one for the horizontal polarization, whose features are detailed in the next section. After the down-conversion, a SPDT switch selects the polarization that is connected to the back-end stage.

Figure 1 summarizes the connections between the antenna set, the front-end stage and the back-end stage.

#### 2.1.3. Back-End Stage

The main purpose of the back-end stage is to measure the power level received at each one of the eight frequency bands, and each polarization of the antenna set. MERITXELL has been designed to perform these measurements using a common power detector, and hence, simplifying the overall design of the radiometer. Furthermore, this back-end stage allows to perform real-time processing, and post-processing of the radiometric signal in order to detect RFI signals, and mitigate them.

**Figure 4:**
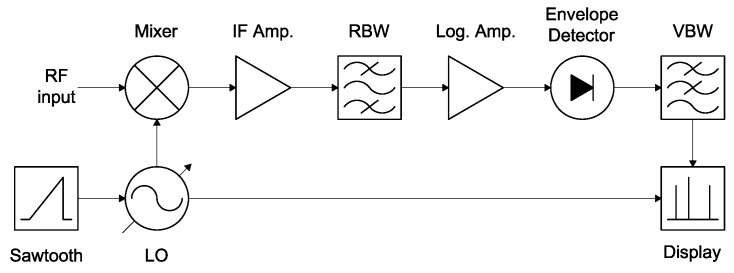
Functional diagram of a common Spectrum Analyzer.

The common power detector stage is completely performed by a Rohde & Schwarz (R&S^®^)FSP40 Spectrum Analyzer (SA) with a frequency range up to 40 GHz (Rohde and Schwarz, Munich, Germany) [26], that allows to process the seven lower frequency bands, from L- to Ka-band. The rationale is that the SA functional block diagram is almost identical to the back-end stage of a passive Total-Power Radiometer (TPR). As can be seen in Figure 4, the signal coming from the front-end stage is first down-converted to IF using a frequency mixer, and a Local Oscillator (LO). Then, the IF signal is amplified and band-pass filtered with the so-called Resolution Bandwidth (RBW). After that, the envelope is detected and low-pass filtered with the so-called Video Bandwidth (VBW). Eventually, the measurement of the power level is displayed or stored for later processing. In addition, when the LO is a variable frequency oscillator, as it is in a SA, the back-end stage becomes a spectroradiometer.

**Figure 5:**
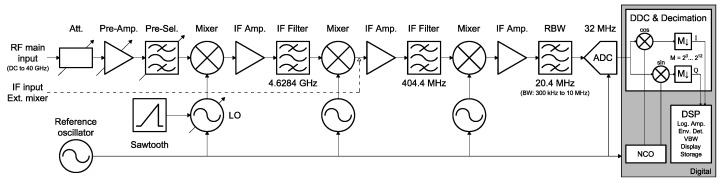
Real implemented architecture of the R&S^®^FSP40 Spectrum Analyzer [26].

However, the real SA architecture is not identical to the functional block diagram. In fact, there are some differences even though the final performance is equivalent. The architecture of the R&S^®^FSP40 is based in a triple superheterodyne down-conversion followed to a digital IF stage as can be seen in Figure 5 [26]. On the one hand, the triple down-conversion overcomes the difficulty of obtaining sufficient selectivity for the higher frequencies, as compared to the former single mixer. On the other hand, the digital IF stage replaces the analog envelope detector and the VBW, by adding versatility and functionality to the final display and storage of the data.

Furthermore, the W-band is treated in a singular way because the R&S^®^FSP40 main port can only reach up to 40 GHz. Therefore, the SA has been equipped with a R&S^®^FSP-B21 external mixer port module which enables it with the capability to receive signals centered at a frequency higher than 40 GHz. In particular, W-band signals can be processed when the SA is combined with the harmonic mixer R&S^®^FS-Z110. This harmonic mixer replaces the first two stages of the triple superheterodyne conversion, and therefore, it down-converts the W-band to a IF located at 404.4 MHz (see Figure 5). From thereafter, the W-band signal is processed in the same way than the other seven frequency bands.

As above mentioned, the architecture of the R&S^®^FSP40 enables the capability of performing real-time processing of the radiometric data in order to detect RFI signals because of the inherent features of a SA. With them, the signal may be divided into several sub-bands that can be weighted to equalize or mimic arbitrary frequency responses of different instruments, time intervals, and calculate histograms and statistic moments of the received signal for each sub-band.

Moreover, the SA has the capability to store directly the In-phase and Quadrature (I/Q) components of the digital IF signal allowing a later post-processing of the radiometric data in the last stage. The post-processing stage is performed in the host computer that can run several RFI detection and mitigation algorithms such as time-frequency blanking, normality tests, or the Multiresolution Fourier Transform (MFT) detailed in [16]. Eventually, the radiometric data can be sent out to the host computer to perform an extra off-line processing.

### 2.2. Additional Sensors

MERITXELL has been equipped with other sensors at different bands, in addition to the radiometer assembly. The data collected by these additional sensors can be sent to the host computer for post-processing together with the radiometric data in order to complement, and to add relevant information to the radiometric data, and at the end to perform data fusion techniques. This data is not send when MERITXELL is neither configured as a conventional radiometer nor in RFI detection applications, and its use for data fusion applications is out of the scope of this work. The additional sensors connected as in Figure 1 and shown in Figure 2, include a GNSS reflectometer, a Thermal Infrared (TIR) camera, a multispectral camera, and a visible camera.

#### 2.2.1. GNSS Reflectometer

GNSS-R can be used for the retrieval of many geophysical variables such as soil moisture [27] and ocean wind speed [28]. Moreover, GNSS-R may be used combined with L-band radiometry in order to correct errors in temperature brightness measurements induced by the characteristics of the measured surface [10]. For this reason, a GNSS-R device using Global Positioning System (GPS) L1 C/A code has been installed in MERITXELL.

The antenna of the GNSS reflectometer consists of an array of 5 Left-Hand Circularly Polarized (LHCP) GPS L1-band (1575.42 MHz) ceramic patches (see Figure 2), with a power combiner that assigns different weights to every patch depending on its position in the array. As in L-, S-, and C-band antennas, Barlett tapering has been applied to reduce the side lobes and improve the main beam efficiency.

The output of the antenna is boosted by means of a tuned amplifier whose output is connected to a SIGE GN3S GPS sampler module [29]. The sampler module is connected by a USB cable to the on-board computer which can be accessed via Ethernet. The on-board computer acts just as a gateway between the internal devices and the host computer. GNSS-R sampled data is finally sent to the host computer for further processing.

#### 2.2.2. Thermal Infrared Camera

One of the optical cameras integrated in MERITXELL is a TIR camera designed to provide thermographic imagery and repeatable temperature measurements. The data obtained with the TIR camera may be used to increase the accuracy of the radiometric measurements since it provides a real-time estimation of the physical temperature.

The TIR camera model is a FLIR A320 camera (FLIR Systems, Wilsonville, OR, USA) [30] with a spectral range from 7.5 μm to 13 μm, a Field-Of-View (FOW) of 25${}^{\circ }$ × 18.8${}^{\circ }$, a detector resolution of 320 × 240 pixels, and a maximum image frequency of 30 frames per second. In addition, the physical temperature can be measured with a thermal sensitivity (i.e., radiometric resolution) of 50 mK at 300 K.

#### 2.2.3. Multispectral Camera

One more sensor is a multispectral camera that has two configurable modes: Red-Green-Blue (RGB) or Color-Infrared (CIR). Multispectral cameras are commonly used in many remote sensing applications. In particular, the CIR configuration is chosen to study vegetation-covered areas because vegetation is highly reflective at Near-Infrared (NIR) band ([31], p. 7); whilst the RGB configuration is preferred in applications such as analyzing man-made objects, performing atmospheric and deep water imaging, or studying vegetation structures.

The multispectral camera model is a DuncanTech MS4100 (DuncanTech, Auburn, CA, USA) [32] with a FOW of 60${}^{\circ }$, a detector resolution of 1920 × 1080 pixels, and a maximum image frequency of 10 frames per second. The camera has 3-CCD image sensors that can work in either in RGB, or in CIR mode, and hence, it can measure electromagnetic radiation at four optical frequency bands: Blue (350–500 nm), Green (500–600 nm), Red (600–700 nm), and NIR (750–850 nm).

#### 2.2.4. Video Camera

The third and last camera is a Wansview NC510 IP camera (Wansview, Shenzhen, China) [33], controlled remotely via an Ethernet connection. This camera adds real-time visible imagery to the scene from where the data is being measured.

### 2.3. Monitoring and Control Systems

In this sub-section, the auxiliary systems needed for thermal stabilization, temperature monitoring, instrument control, and data retrieval are introduced. All the information retrieved from these sub-systems, and the necessary commands to control them are sent to and received from an external host computer via Ethernet connection.

#### 2.3.1. Enclosure

**Figure 6:**
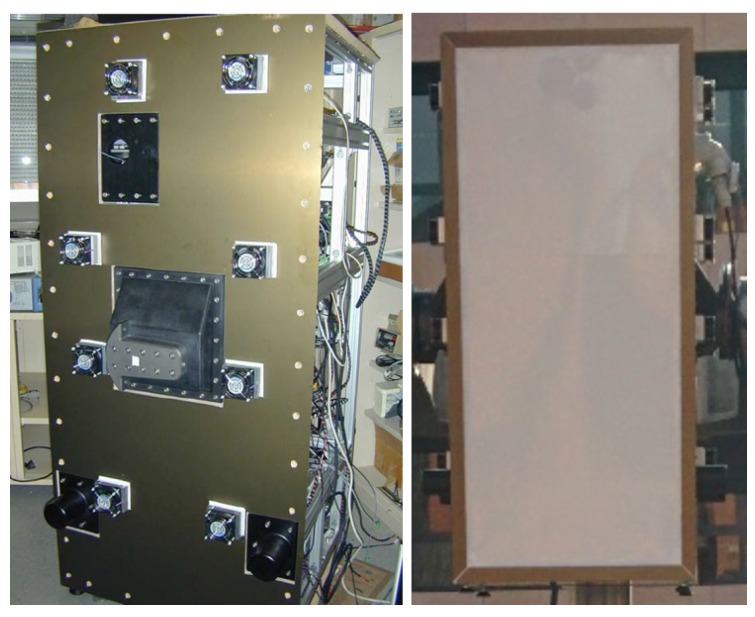
Side view of MERITXELL’s enclosure (**Left**); and front view the antenna radome (**Right**).

Thermal stabilization is an essential need of a microwave radiometer, since variations of the physical temperature will lead to variations in the measurements. The origin of these variations is the dependence on the physical temperature of the gain and noise figure of all the elements of the radio-frequency chain. Consequently, the radiometer must be thermally insulated. This is achieved using an enclosure designed to minimize the heat exchange between inside and outside the radiometer. The walls of MERITXELL are 20 mm dual-side metallized foam boards (both for electromagnetic interference and thermal insulation), except for the front-part. The antennas are covered with a 5.5 mm thick Depron^®^ radome (a kind of white polystyrene foam) showing losses of approximately 0.25 dB at W-band. The metallic part of the walls is composed of two sheets of 1 mm thickness aluminum. Both the metallic enclosure, and the white radome can be seen in Figure 6, with approximate dimensions of 180 cm × 90 cm × 90 cm.

#### 2.3.2. Thermal Stabilization

Microwave radiometers usually work at a constant temperature higher than the outside so that the maximum antenna temperature (with no RFI signals) will be eventually lower than the internal temperature. Moreover, it is easier to perform the temperature stabilization by using heating elements. Hence, MERITXELL radiometer is designed to work at a constant temperature around 45 ${}^{\circ }$C.

The temperature control is performed using a 2216 L Eurotherm Proportional Integral Derivative (PID) temperature controller (Eurotherm, Worthing, UK).The temperature inside the radiometer is measured with a PT100 temperature dependent resistance. With this temperature measurement, the PID is able to maintain the temperature inside the radiometer at a determined constant value with the control of a set of heaters and Peltier cells.

#### 2.3.3. Thermal Monitoring

In order to measure periodically the temperatures of the matched loads and the amplifiers of each radiometric band and polarization, 32 DS18B20 digital temperature sensors (one for each of the 16 amplifiers and 16 matched loads, Maxim Integrated, CA, USA) are used.These sensors provide temperature measurements with an accuracy of ±0.5 ${}^{\circ }$C. The sensor readings are controlled by a microcontroller which make the data available to the host computer via an Ethernet connection. Then, once the temperature values are retrieved, they can be used to calibrate and correct the radiometric measurements.

#### 2.3.4. Switch Control

As mentioned above, the switches and latching circulators select the polarization and the radiometric input (antenna or matched load) for the different frequency bands. A microcontroller with Ethernet connection allows to control from the host computer the 6 switches and the 10 latching circulators that determine the radiometric input, and the 2 switches that select the polarization (see Figure 1).

### 2.4. Mobile Unit

As above mentioned, MERITXELL dimensions are approximately 180 cm × 90 cm × 90 cm, and the total weight is larger than 250 kg. These weight and dimensions make the handling of MERITXELL a very complex task. Therefore, a mobile unit was designed and manufactured by an external company in order to transport and handle the positioning of MERITXELL.

#### 2.4.1. Telescopic Robotic Arm

**Figure 7:**
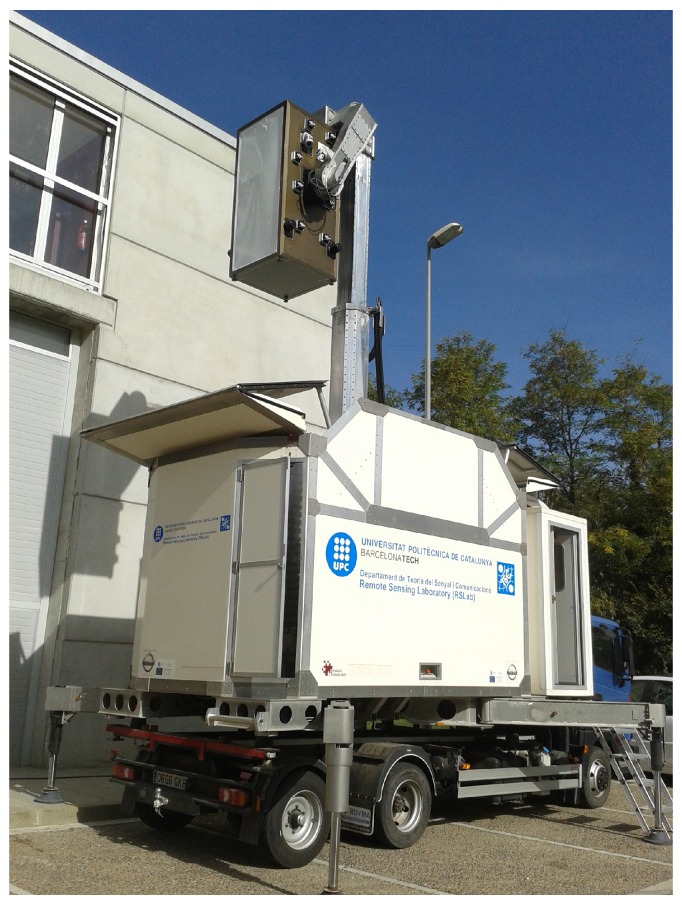
MERITXELL assembled to the telescopic robotic arm above the housing structure and the truck.

This mobile unit consists of a telescopic robotic arm able to handle the radiometer and to manage its positioning, mounted on a NISSAN ATLEON truck that allows to transport the entire system. In order to position the radiometer, the arm is capable to rise it up to 8 m from the ground, and then to change its pointing direction using two different rotors. One controls the azimuth of MERITXELL. Azimuth can be set from 45${}^{\circ }$ to 315${}^{\circ }$, where 0${}^{\circ }$ is pointing to the cabin of the truck, and the angle increase in counterclockwise direction. The other rotor controls the elevation movement, defined as the zenith angle, where it can be modified from 0${}^{\circ }$ (zenith) to 155${}^{\circ }$ (incidence angle of 25${}^{\circ }$). Both angle restrictions are set for safety reasons. Moreover, the telescopic arm has three states: up or measuring, down or parked, and calibration or pointing to a microwave absorber, which is used to perform the hot load calibration. All the structure is mounted inside a fiber-glass housing for transportation and storage purposes. Figure 7 shows MERITXELL assembled to the telescopic arm above the housing structure and the truck.

Moreover, the mobile unit has four stabilization legs manually controlled covering the maximum surface allowing to work with an instrument at eight meters high withstanding winds of up to 100 km/h. Both the telescopic robotic arm and the stabilization legs work with an hydraulic unit.

#### 2.4.2. Positioning Control

The telescopic robotic arm is controlled by a Programmable Logic Controller (PLC) located in a control panel inside the truck. This PLC is connected to the host computer via a serial connection, which sends the commands needed to move MERITXELL radiometer to the desired position.

## 3. Instrument Control

The positioning, configuration, and operation of MERITXELL is controlled from a dedicated software running on the host computer. The host computer sends and receives the desired commands to MERITXELL via Ethernet connection, whereas the communication with the PLC of the positioning system takes place via serial port.

MERITXELL software has been designed to perform the radiometric measurements, including the selection of the frequency band and bandwidth to store the retrieved data into the host computer, and to manage the handling and positioning of the radiometer. Besides, the configuration and data retrieval of the additional sensors is done using their dedicated software programs. However, the positioning functionality of the presented MERITXELL software can still be used for the additional sensors (e.g., cameras).

### 3.1. Graphical User Interface

**Figure 8:**
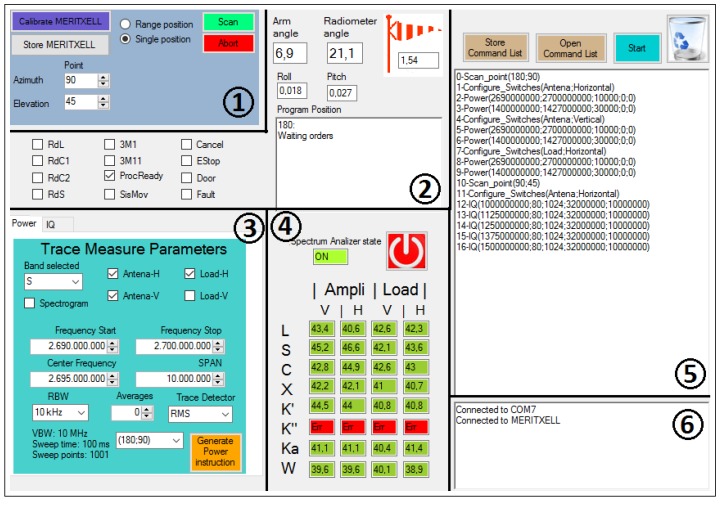
Graphical user interface of the host program used to control MERITXELL. It contains six different parts: (**1**) positioning control; (**2**) positioning monitoring; (**3**) measurement control; (**4**) temperature monitoring; (**5**) command list; and (**6**) debugger.

MERITXELL software has a user-friendly Graphical User Interface (GUI) that allows to display the information received from MERITXELL and its positioning system, and to generate the desired configuration instructions. The operation of the software is based on the generation of a list of command instructions which are executed sequentially. The possible commands comprise configuration of the spectrum analyzer, switch control, and positioning instructions. Figure 8 shows the GUI of MERITXELL software, which is divided into six blocks described below.

Block 1 allows to generate the instructions related to the positioning of MERITXELL. First of all, the instrument can be set either to the store position or to the calibration position with the corresponding buttons and located inside the housing enclosure. At the calibration position, the antenna set is in front of a microwave absorber with is used as hot load for calibration purposes. Moreover, the scan option is used to point the instrument to either a specific direction or a range of positions defined by their azimuth and elevation. In order to satisfy the security restrictions, the software does not allow to generate pointing values with an azimuth lower than 45${}^{\circ }$ or larger than 315${}^{\circ }$, and an elevation greater than 155${}^{\circ }$ (recall that the elevation is defined as the zenith angle). Eventually, the abort button allows the user to immediately retract the robotic arm, and to store the instrument.

Block 2 displays several parameters related to the current state of the positioning system. The arm and radiometer angles determine the current position of the two axes of the robotic arm that holds the instrument. The former is the angle between the mast and the rotation axis of MERITXELL, whereas the latter is the current elevation angle determined by the encoder of the rotation axis. In addition, the roll and pitch of the overall structure, together with the wind speed (red flag in Figure 8) measured with an anemometer at the top of the mast, are used to monitor if the positioning system is working under safe conditions. Furthermore, the rest of the elements of Figure 8 block 2 contain additional information related to the operation of the positioning system.

Block 3 generates the instructions to configure the back-end (the spectrum analyzer), and the matched load and polarization switches. The back-end can be configured to retrieve either the spectrum at the selected band, or a spectrogram (a collection of consecutive spectra), or directly sampled I/Q signals. Further details of each kind of measurement are provided in the subsequent section. Moreover, each one of these measurements can be attached to one or more scanning positions generated using Block 1. Eventually, this block also allows the user to select between measurements taken from the antenna or from the matched load, and at either horizontal or vertical polarization.

Block 4 displays the values of the temperature sensors located at the matched loads and amplifiers of each frequency band. These values are the average of the last ten temperature measurements of each sensor. This gives a resolution of 0.02 K according to the temperature sensor specifications. Moreover, the gradient of each temperature value is represented using a color code being red if the gradient is higher than 3 mK/s, yellow if it is higher than 1.5 mK/s, blue if it is lower than −1.5 mK/s, and green otherwise. These values are obtained dividing the difference between the average of the last and the previous fifteen measurements by the mean time between the averages which is 37.5 s. The green color is used to indicate that the system is stable in temperature (±1.5 mK/s), and, if this is the case, the desired measurements can be performed. Eventually, temperature values are stored once a measurement takes place for further processing.

Block 5 shows the list of instructions to be executed. These are generated using Block 1 and Block 3, and they are executed when the "Start" button is pressed. The instructions are executed sequentially in the order of appearance, and the program runs until all of them are done. Furthermore, the list of instructions can be saved and loaded for further executions.

Block 6 shows messages that help the user to follow the execution process, and to debug possible errors.

### 3.2. Back-End Configuration

As mentioned above, the back-end stage of the multiband radiometer is implemented using a spectrum analyzer R&S^®^FSP40. The configuration of the SA is implemented using several built-in commands sent via Ethernet connection. Block 3 of the GUI software helps the user to generate these configuration commands to get the measurements and retrieve the data. There are two modes of operation: power or I/Q measurements.

#### 3.2.1. Power Measurements

The power mode allows to get directly the power spectral density of the system noise measured by the SA. When several noise spectra are retrieved consecutively, their orderly aggregation conforms a spectrogram. The spectrogram allows to detect temporal changes in the spectrum, however, it requires larger communication bandwidth and acquisition time. Each point of the spectrum represents the radiometric noise power spectral density corresponding to a specific frequency in the span bandwidth, with a noise bandwidth equal to the resolution bandwidth of the SA. In order to understand clearly this last statement, the configurable SA parameters and their relationship with the radiometric performance are described subsequently.

The *center frequency* $f_{c}$ determines the central point of the frequency band under analysis. According to the front-end design, there are eight usual values for this parameter, which are the center frequency of each band.

The *span* $B_{r}$ is the overall receiving bandwidth to be analyzed centered at $f_{c}$. As the previous parameter, it is usually defined as the bandwidth of the eight frequency bands supported by the front-end. The *resolution bandwidth* RBW is the bandwidth of the last and narrower bandpass filter in the IF chain (see Figure 5). This value determines the noise power and resolution of each point of the spectrum. The possible values for RBW are increasing from 10 Hz to 10 MHz in 1, 3, 10 steps [26].

The *video bandwidth* VBW is the bandwidth of the lowpass filter set after the detector. For radiometric purposes, this value is automatically set to ten times the resolution bandwidth, VBW=10×RBW, in order to ensure that the peak values of the voltage after the detector are not cut off by the video filter [26].

The *sweep time* SWT is the time that the local oscillator takes to sweep the whole $B_{r}$. This parameter is automatically set to its minimum value [26]

(1) $$\mathrm{SWT}=k\frac{B_{r}}{{\mathrm{RBW}}^{2}},$$

where k is a is a constant internal parameter of the R&S^®^FSP40.

The *number of sweep points* $N_{\mathrm{SWP}}$ is the number of points obtained for each spectrum measurement. Given the SA structure, samples are statistically independent if the time difference between consecutive acquisitions is at least 1/RBW. This means that the maximum number of statistically independent samples $N_{\mathrm{IND}}$ taken for each $B_{r}$ is equal to

(2) $$N_{\mathrm{IND}}=\frac{B_{r}}{\mathrm{RBW}}.$$

However, $N_{\mathrm{SWP}}$ can only be configured as one of the values of the set 8001, 4001, 2001, 1001, 501, 251, and 125. Therefore, this parameter is set automatically by the SA to the minimum value of the previous set that satisfies the inequality

(3) $$N_{\mathrm{SWP}}>\frac{B_{r}}{\mathrm{RBW}}.$$

The *trace detector* determines the algorithm used in the detection process. There are three types of detectors: *sample* which uses a midpoint value for each sweep point, *peak* which uses the maximum or minimum detected value for each point, and *average* which uses all of the detected values within a point to calculate the final value. The default algorithm used in MERITXELL is the average of power using the Root Mean Square (RMS) of the input samples.

The power spectrum retrieved with the RMS detector contains $N_{\mathrm{SWP}}$ measurements of the radiometric noise in the whole bandwidth under analysis $B_{r}$. The measured power value for each point of the spectrum is

(4) $$P_{\mathrm{RMS}}\left(f_{p}\right)=\frac{1}{N_{p}}\sum_{m=0}^{N_{p}-1}{\left|s\left[m+n_{\mathrm{SWP}}\;N_{p}\right]\right|}^{2},$$

where $n_{\mathrm{SWP}}\in \left[0,1,\cdots ,N_{\mathrm{SWP}}-1\right]$ is the index of each sweep point, $f_{p}=f_{c}-\frac{B_{r}}{2}+n_{\mathrm{SWP}}\frac{B_{r}}{N_{\mathrm{SWP}}-1}$ is the frequency of each point of the spectrum, s[m] is the linear digitized video voltage at the output of the Analog-to-Digital Converter (ADC) at the SA, and $N_{p}$ is the number of values of the ADC per sweep point defined as

(5) $$N_{p}=F_{s}\frac{\mathrm{SWT}}{N_{\mathrm{SWP}}}=F_{s}\;T_{\mathrm{SWP}},$$

where $F_{s}$ is the sampling frequency of the ADC (32 MHz), and $T_{\mathrm{SWP}}$ is the sweep time per sweep point.

The *number of averages* $N_{\mathrm{AVG}}$ is the number of sweeps or averaged spectra per measurement. The number of averages increases proportionally the integration time. If the spectrogram option is active, $N_{\mathrm{AVG}}$ determines the number of consecutive spectra. Besides, if $N_{\mathrm{AVG}}$ is set to either 0 or 1, only one sweep is performed. The averaged spectrum can be expressed as

(6) $$\bar{P_{\mathrm{RMS}}}\left(f_{p}\right)=\frac{1}{N_{\mathrm{AVG}}}\sum_{i=0}^{N_{\mathrm{AVG}}}P_{{\mathrm{RMS}}_{i}}\left(f_{p}\right),$$

whereas if the value of all spectrum points are averaged, the measured radiometric power over the acquisition band $B_{r}$ can be defined as

(7) $$P_{m}=\frac{1}{N_{\mathrm{SWP}}}\sum \bar{P_{\mathrm{RMS}}}\left(f_{p}\right).$$

Taking into account the parameters mentioned above and the resolution of a TPR ([1], pp. 359–366), the radiometric resolution of MERITXELL multiband radiometer per sweep point using the RMS detector, and several averaging points is

(8) $$\Delta P_{\mathrm{RMS}}\left(f_{p}\right)=\frac{\bar{P_{\mathrm{RMS}}}\left(f_{p}\right)}{\sqrt{\mathrm{RBW}\;T_{\mathrm{SWP}}\;N_{\mathrm{AVG}}}}=\frac{\bar{P_{\mathrm{RMS}}}\left(f_{p}\right)}{\sqrt{\mathrm{RBW}\frac{\mathrm{SWT}}{N_{\mathrm{SWP}}}N_{\mathrm{AVG}}}}.$$

Eventually, the resolution may be increased when averaging all the points of the spectrum. However, since there may be an overlapping of the RBW from consecutive sweep points ($N_{\mathrm{SWP}}\geq N_{\mathrm{IND}}$), the overall radiometric resolution after averaging all sweep points is

(9) $$\Delta P_{m}=\frac{P_{m}}{\sqrt{N_{\mathrm{IND}}\;N_{\mathrm{AVG}}}}=\frac{P_{m}}{\sqrt{\frac{B_{r}}{\mathrm{RBW}}\;N_{\mathrm{AVG}}}}.$$

#### 3.2.2. I/Q Measurements

**Figure 9:**
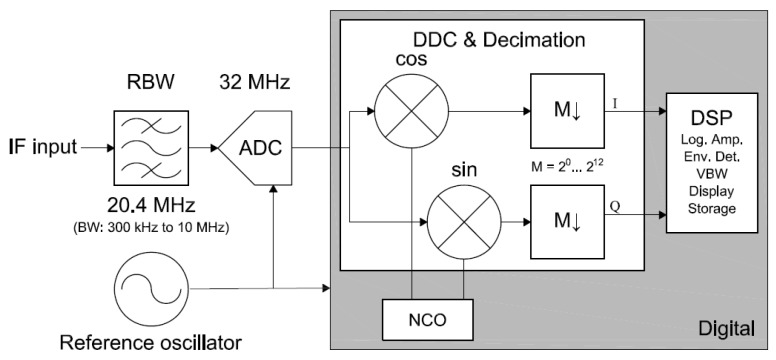
Diagram of the digital downconversion and sample retrieval of the I/Q acquisition mode.

The I/Q acquisition mode allows to retrieve directly the in-phase and quadrature components of the radiometric signal. This mode is needed for advanced RFI detection and mitigation techniques in the host computer. In this mode, the radiometric signal is conditioned as in power mode until it reaches the ADC (see Figure 5). Once the signal is digitized, the signal is downconverted to its baseband I/Q components, which are stored and sent to the host computer for further processing. Figure 9 shows a diagram of the digital downconversion structure.

Using the I/Q mode, the SA configuration parameters take a slightly different meaning as compared to the power mode:

- The *central frequency* configures the frequency of the local oscillator (external mixer in the W-band), which is fixed in this mode.
- The *reference level* determines the maximum amplitude (or power) of the dynamic range of the ADC.
- The *ADC filter* sets the bandwidth of the anti-aliasing filter before the ADC. It is equivalent to the RBW, but its possible values are 10 MHz, 3 MHz, 1 MHz, and 300 kHz.
- The *sample rate* sets the decimation value after the digital downconversion, and then the output sample rate. The decimation value increases in powers of 2 from 1 to 2048.
- The *number of samples* determines the size of each data acquisition. The available buffer can store is up to 128 k samples.

## 4. Calibration and Characterization

This section describes the measuring principle, the radiometric stability, and the calibration procedure and results of the multiband dual-polarization total power radiometer of the instrument MERITXELL.

### 4.1. Measuring Principle

The goal of a radiometer is to measure the antenna temperature $T_{A}$, which is defined as the radiometric noise power collected by the antenna. However, since all the antennas have some losses, the antenna temperature measured at its output terminals $T_{A}^{\prime }$ is ([1], pp. 207–208)

(10) $$T_{A}^{\prime }=\eta_{A}\;T_{A}+\left(1-\eta_{A}\right)T_{pA},$$

where $\eta_{A}$ is the antenna radiation efficiency, and $T_{pA}$ is the physical temperature of the antenna. Approximate values for $\eta_{A}$ are shown in Table 2. Signals received by the antennas are amplified and filtered by the front-end and back-end stages, which also introduce some additional noise. Therefore, the power level measured at the input of the detector $P_{R}$ is ([1], pp. 350–352)

(11) $$P_{R}=k_{B}\;G_{r}\;T_{sys}\;B_{r}=k_{B}\;G_{r}\left(T_{A}^{\prime }+T_{r}\right)B_{r},$$

where $k_{B}$ is the Boltzmann constant, $G_{r}$ is the total gain of the receiving chain, $T_{sys}$ is the total system temperature, $T_{r}$ is the equivalent input noise temperature of the receiving chain, and $B_{r}$ is the system bandwidth.

In the case of MERITXELL, because of the high gain of the first stage (more than 60 dB), $T_{r}$ can be approximated by the equivalent noise temperature introduced by the front-end stage, and the $B_{r}$ is set by the bandwidth of the back-end stage (span parameter of the SA).

After that, the noise signal reaches the detector, and the power level measured at its output $P_{m}$ can be expressed after combining previous equations as

(12) $$P_{m}=G_{d}\;P_{R}+P_{d}=G_{d}\;G_{r}\;k_{B}\left(\eta_{A}\;T_{A}+\left(1-\eta_{A}\right)T_{pA}+T_{r}\right)B_{r}+P_{d},$$

where $G_{d}$ is the conversion gain, and $P_{d}$ accounts for a possible bias of the detector.

In order to determine the number of unknown parameters in (12), and hence, to known the equivalence between the antenna temperature and the measured power after the detector, a calibration procedure must be applied.

### 4.2. Calibration Procedure

According to (12), the power measured by a radiometer is a linear function of the antenna temperature, and a number of a priori unknown parameters. The calibration process is required to obtain the values of these parameters, which vary with temperature and frequency band. As detailed in Section 2.3.2, thermal stabilization is of crucial importance in this case, in order to keep constant the values of the unknown parameters, and in particular, the gain of the amplifiers.

Two different calibration methods are used depending on the frequency band. In the case of the three lower bands (L-, S-, and C-band), the simple hot-cold calibration ([1], pp. 402–404) is applied using the microwave absorber at a known temperature set in front of the antennas as hot load, and the radiometer pointing to the sky at zenith position as cold load. On the other hand, the upper bands are calibrated combining the tipping curves method [34,35], which is used to retrieve several cold calibration points, with the hot-cold method. Calibration using tipping curves cannot be applied at the L-, S-, and C-band because of the wide antenna pattern and its non-negligible side lobes, which start collecting radiation from ground when the beam is not pointing to the zenith.

#### 4.2.1. Hot-Cold Calibration

The hot-cold calibration is the simplest method used in calibration of microwave radiometers. It is based in a two point linear approximation of the measured power as a function of the antenna temperature. One point corresponds to the power $P_{hot}$ and antenna temperature $T_{abs}$ measured at the hot calibration point (looking to the microwave absorber), and the other corresponds to the power $P_{cold}$ and antenna temperature $T_{sky}$ measured at the cold calibration point (looking to the sky at zenith). The hot-cold calibration can be modeled as the linear system of equations

(13) $$\left.\begin{array}{c} P_{hot}=a\;T_{abs}+b\\ P_{cold}=a\;T_{sky}+b \end{array}\right\}$$

where

(14) $$a=G_{d}\;G_{r}\;k_{B}\;\eta_{A}\;B_{r},$$

and

(15) $$b=\frac{a}{\eta_{A}}\left(\left(1-\eta_{A}\right)T_{pA}+T_{r}\right)+P_{d}.$$

The physical temperature of the microwave absorber can be obtained using either an external sensor, or MERITXELL’s TIR camera. Besides, the zenith sky temperature can be approximated by the temperature of the cosmic background plus the downwelling atmospheric temperature ($T_{sky}∼$ 6 K), which are nearly independent of the atmospheric conditions between 1 and 10 GHz ([1], p. 287).

Therefore, measuring the values of $P_{hot}$ and $P_{cold}$, *a* and *b* can be obtained from (13) as

(16) $$a=\frac{P_{hot}-P_{cold}}{T_{abs}-T_{sky}},$$

and

(17) $$b=\frac{P_{cold}\;T_{abs}-P_{hot}\;T_{sky}}{T_{abs}-T_{sky}}.$$

Once the *a* and *b* parameters are obtained for each band, the relationship between the output and the input of the radiometer is fully characterized. In addition, the power level $P_{load}$ measured when the switch is commuted to the matched load can be expressed using (14) and (15) as

(18) $$P_{load}=\frac{a}{\eta_{A}}\left(T_{load}-\left(1-\eta_{A}\right)T_{pA}\right)+b.$$

Equation (18) can be also used for the hot-cold calibration, only if $T_{load}=T_{pA}$. In MERITXELL, the identity $T_{load}=T_{pA}$ is fulfilled to within $\pm 0.5^{\circ }$C thanks to the thermal insulation and stabilization sub-systems.

#### 4.2.2. Tipping Curves

The tipping curves are a series of measurements of the sky at different zenith angles, which are adopted for convenience in the calibration of ground-based radiometers, assuming a horizontally stratified atmosphere. With the information of the sky opacity, it is possible to determine the brightness temperature of the sky at any zenith direction which can be used as a cold calibration target [34,35].

Taking into account the radiative transfer equation under the Rayleigh-Jeans approximation and the assumption of a single layer atmosphere, the antenna temperature under a zenith angle θ is given by

(19) $$T_{A}\left(\theta \right)=T_{cos}\;e^{-\tau (\theta )}+T_{m}\left(\theta \right)\left(1-e^{-\tau (\theta )}\right),$$

where $T_{cos}$ is the cosmic background radiation (∼2.7 K), $T_{m}\left(\theta \right)$ is the average temperature of the troposphere, and τ(θ) is the total slant opacity. According to [34], the slight angular dependence of $T_{m}\left(\theta \right)$ can be ignored for low opacity channels such as the ones selected for MERITXELL, and its value can be estimated from the ground temperature $T_{gr}$ as $T_{m}\left(\theta \right)\approx T_{m}\left(0\right)\approx T_{gr}-10$ K. Moreover, the total slant opacity can be defined as

(20) τ(θ)=τ(0)sec(θ),

where sec(θ) is equivalent to the number of atmospheres that contribute to the antenna temperature, and then, from (19)

(21) $$T_{A}\left(\theta \right)=T_{m}+\left(T_{cos}-T_{m}\right){\left(L_{atm}\right)}^{\sec(\theta )},$$

where $L_{atm}=e^{-\tau (0)}$ is the total atmosphere attenuation at zenith direction.

Equation (21) defines the tipping curves for a ground-based microwave radiometer, which allow to determine the atmospheric opacity at zenith τ(0), and then to obtain the $T_{A}\left(\theta \right)$ values to be used as cold temperature points in the hot-cold calibration. Eventually, (21) can be combined with the hot-cold calibration procedure (13), in order to obtain the tipping cures in terms of measured power as

(22) $$P_{cold}\left(\theta \right)=a\left(\left(T_{m}-T_{hot}\right)+\left(T_{cos}-T_{m}\right){\left(L_{atm}\right)}^{\sec(\theta )}\right)+P_{hot}.$$

### 4.3. Calibration Examples

In this subsection, the results of one example of each of the calibration procedures mentioned in the previous section are shown. Measurements have been performed in clear sky conditions, after the sunset, at a ground temperature $T_{gr}$ of 9 ${}^{\circ }$C, and with the receiving chain stabilized in temperature.

The first example corresponds to the calibration of the C-band V-polarization channel. In this case, six calibration points are measured, two hot ones pointing to the microwave absorber, two cold ones pointing to the sky at zenith, and two matched load measurements. The measurements have been taken using the following parameters: the acquisition band $B_{r}$ is 90 MHz, the RBW is 100 kHz, the $N_{\mathrm{SWP}}$ is 1001, the SWT is 9 ms, and the $N_{\mathrm{AVG}}$ is 1000.

**Figure 10:**
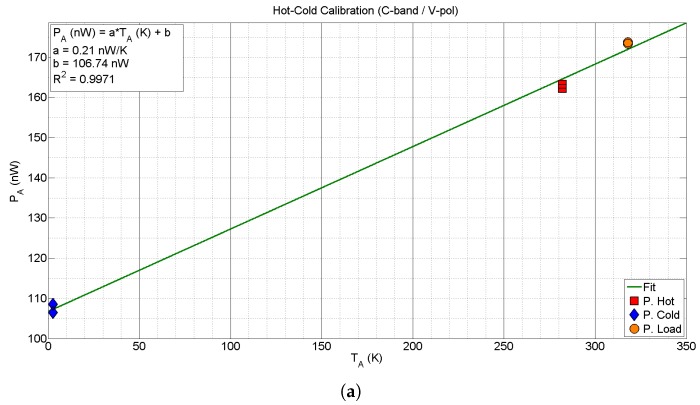

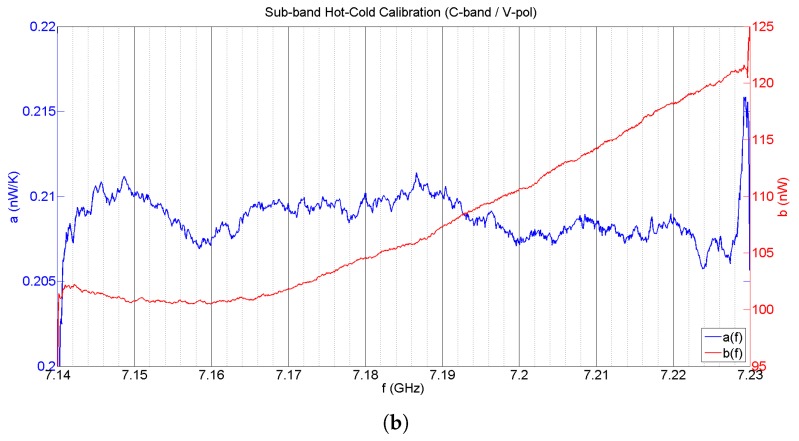
Hot-cold calibration of the C-band V-polarization channel. Subfigure (**a**) shows the calibration procedure for averaged values in the whole bandwidth; whereas (**b**) depicts the *a* and *b* values obtained for each sub-band.

Using these measurements, the *a* and *b* parameters are obtained by solving (14) and (15) using a linear regression method. Since the back-end stage provides power spectrum measurements, *a* and *b* parameters may be obtained either by averaging the whole spectrum or for particular sub-bands. If the power spectrum is averaged over the whole acquisition bandwidth, the values obtained are a=0.21 nW/K, and b=106.74 nW, with a $R^{2}$ parameter (i.e., coefficient of determination) equal to 0.9971. The results of the averaged hot-cold calibration for the C-band V-polarization are shown in Figure 10a. On the other hand, Figure 10b shows the *a* and *b* values for sub-bands equal to the RBW. In this case, it can be appreciated that the gain *a* fluctuates around its average value, and the bias *b* slightly increases with frequency. Moreover, the band-limiting effect of the filters generates outlier *a* values (minimum and maximum peaks) at the band edges.

The second example corresponds to the calibration of the X-band H-polarization channel. In this case, thirteen calibration points are measured, two hot ones pointing to the microwave absorber, nine cold ones pointing to the sky at different zenith angles, and two matched load measurements. The measurements have been taken using the following parameters: the acquisition band $B_{r}$ is 100 MHz, the RBW is 100 kHz, the $N_{\mathrm{SWP}}$ is 1001, the SWT is 9 ms, and the $N_{\mathrm{AVG}}$ is 1000.

**Figure 11:**
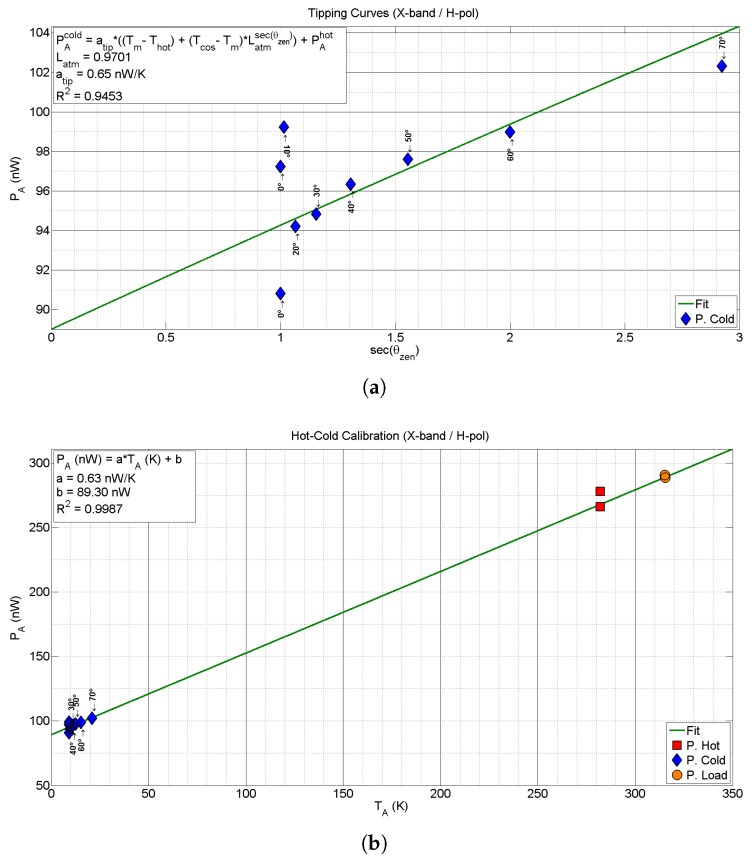
Hot-cold calibration of the X-band H-polarization channel using tipping curves. Subfigure (**a**) shows the power tipping curves as a function of the equivalent number of atmospheres. The elevation angle has been swept from 0${}^{\circ }$ (zenith) to 70${}^{\circ }$, in steps of 10${}^{\circ }$; In subfigure (**b**) the hot, cold and load calibration points are depicted. The cold calibration points have been obtained using the previous tipping curves procedure.

The cold calibration points have been obtained by changing the elevation angle of the radiometer from the 0${}^{\circ }$ (zenith) to 70${}^{\circ }$, in steps of 10${}^{\circ }$, and one extra measurement has been acquired at the zenith position. This corresponds to values from one to approximately three equivalent atmospheres. Figure 11 shows the results of the tipping curve equation defined in (22) for the X-band H-polarization channel, as a function of the equivalent number of atmospheres (secant of the elevation angle). With these values and using a robust (outlier values were discarded) non-linear regression method, the total atmosphere attenuation at zenith direction $L_{atm}$ has been estimated to be 0.9701, with a $R^{2}$ parameter equal to 0.9453. Outlier values measured at lower zenith angles are induced by the small (but enough) internal temperature differences that occur when changing the position of the radiometer, despite internal fans are installed to minimize this effect.

Once estimated the $L_{atm}$, the hot-cold calibration can be performed using several cold points to determine the unknown parameters. The results of the hot-cold calibration for the X-band averaged in the whole bandwidth are shown in Figure 11. The *a* and *b* parameters are obtained as in the previous case. The obtained values are *a* equal to 0.63 nW/K, and *b* equal to 89.30 nW, with a $R^{2}$ parameter equal to 0.9987. Note that in the tipping curves regression the estimation of the a=0.65 nW/K was also very similar.

### 4.4. Radiometric Stability

Once the calibration is performed, the radiometric stability of the receiving chain has been studied. The radiometric stability is the parameter that provides information about how the calibration parameters will drift with time. The radiometric stability depends on several parameters such as temperature drifts, or instability of amplifiers gain, among others [36].

The radiometric stability is calculated as the Allan variance of the retrieved power for each band and polarization [36]. Moreover, it also determines the optimum integration time to achieve the best radiometric sensitivity.

**Figure 12:**
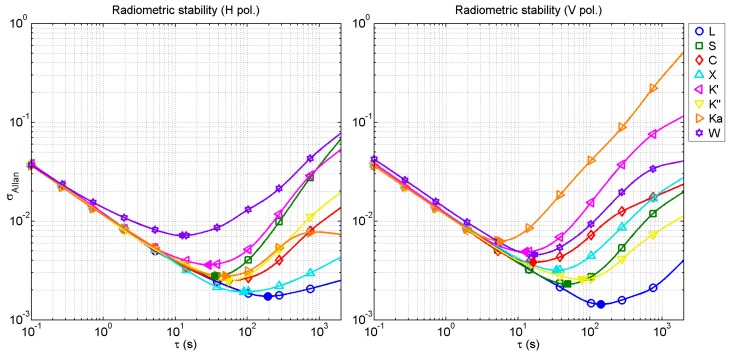
Radiometric stability obtained as the Allan variance for each frequency band and polarization (H **left** and V **right**). Filled markers represent the minimum value for each case, i.e., the optimal integration time.

The Allan deviation measurements (square root of the variance) for all the bands are shown in Figure 12. In addition, the optimum integration time, and its corresponding radiometric resolution are obtained as the minimum of the Allan deviation. These results are shown in Table 3.

Results show that radiometric stability depends on the band under assessment. However, as a general trend, it can be observed that radiometric stability is higher for lower frequency bands since they have larger optimum integration times and lower Allan deviation values.

## 5. RFI Measurements

### 5.1. RFI Related Capabilities

One of the main objectives of instrument MERITXELL is to be a flexible platform to perform experiments related to the detection, characterization, and localization of RFI signals present at the frequency bands used for passive microwave radiometry. In order to avoid self-interference, data buses have been check to not emit any electromagnetic emission in the working bands. This was done during the assembly process of MERITXELL.

According to the designed structure of its back-end, MERITXELL allows to perform real-time RFI signal detection and characterization in time, frequency and statistical domains. The power measurements allow to retrieve the spectrum of the signal under analysis, and thus, to detect RFI signal whose power is more concentrated around some frequency points of the receiving bandwidth. Moreover, thanks to the spectrogram capabilities, RFI signals can be analyzed both in time and frequency domains by identifying temporal changes in the signal spectrum.

Furthermore, the back-end of MERITXELL also allows to perform a statistical analysis of the received signals. As shown in [18], several normality tests can be used to detect the presence of RFI signals, although the combination of the Kurtosis and the Anderson-Darling tests leads to an optimum combo test without blind spots. MERITXELL allows to perform this analysis using the I/Q measurement mode, and then applying such normality tests on post-processing in the host computer. In [21], a statistical analysis of RFI signals was alredy performed using MERITXELL.

### 5.2. Receiving Chain Compensation

Given the architecture of MERITXELL, the spectrum retrieved by the back-end stage is a combination of the received signal spectrum, or the spectrum under analysis, and the frequency response of the receiving chain, including the antenna and front-end stage. The non-ideal frequency response of the combination of antenna, tuned filters, and amplifiers may mask the detection of weak RFI signals. However, the frequency response corresponding to the front-end and back-end stages can still be compensated or equalized by using the spectrum of the corresponding matched load. This compensation is performed dividing point by point the power spectrum taken at the antenna by the power spectrum measured at the matched load. The result of this operation gives a measurement of the relative power of the RFI signal after the antenna over the thermal noise present at the matched noise.

### 5.3. RFI Examples at MWR Bands

It is known that RFI signals are a common problem in MWR bands, and MERITXELL is an outstanding platform to study and characterize them. For this reason, it is not difficult to capture some RFI signals in the majority of the bands covered by MERITXELL, specially in urban environment. The measurements presented hereinafter have been performed at the UPC Campus Nord in Barcelona, Spain. They have been taken at both V and H polarizations, using the spectrogram feature, and the frequency response compensation has been applied. The color scale represents the power spectral density measured at the detector obtained dividing the value of the power spectrograms by the corresponding RBW used in each case (1 MHz for L-, S-, and C-band, and 10 MHz for the others).

**Figure 13:**
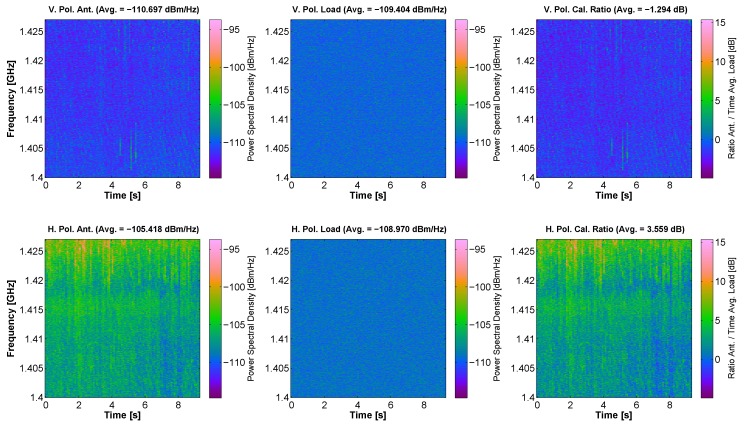
Capture of RFI signals at L-band. The upper subfigures correspond to V polarization, and the lower ones to H polarization. From left to right, the subfigures correspond to spectrograms taken at the antenna, at the matched load, and after the compensation. The spectrograms corresponding to the matched load are relatively flat as compared to the RFI signals captured during the measurement process.

One of the most contaminated bands is L-band. Figure 13 shows a capture of some RFI signals at L-band. The measurements show that at V polarization, some band-limited pulsed signals can be appreciated. Most likely, this kind of RFI signals at L-band are spurious pulses from near-band radar signals. Besides, the behavior at H polarization is even worse in this measurement. This is presumable due to the presence of second harmonics of the terrestrial broadcasting television service, which are emitted at H polarization. Moreover, the measurements at V and H polarizations have not been taken simultaneously because of the inherent structure of MERITXELL. Therefore, if the RFI signal has some temporal variations, they may not appear in both measurements. Furthermore, the spectrograms corresponding to the matched load show that the frequency response of the L-band receiving chain is relatively flat as compared to the RFI signals captured during the measurement process.

**Figure 14:**
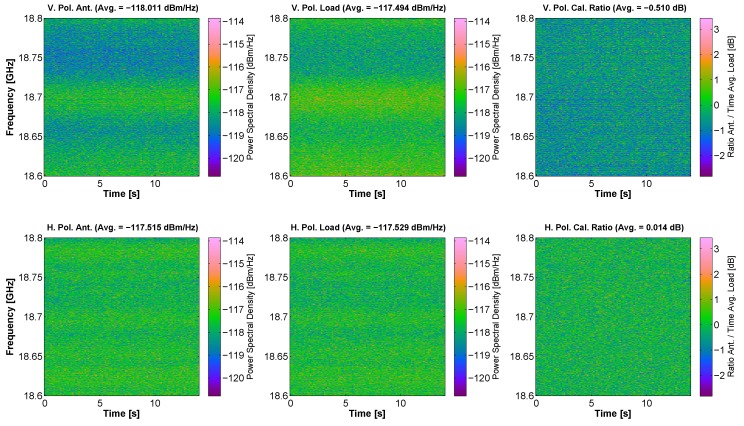
Capture of RFI signals at K’-band. The upper subfigures correspond to V polarization, and the lower ones to H polarization. From left to right, the subfigures correspond to spectrograms taken at the antenna, at the matched load, and after the compensation. The spectrograms corresponding to the matched load show some variations in the frequency response of the receiving chain.

On the contrary, K’-band has some variations in the frequency response of the receiving chain that are on the order of the variations of the received signals. This phenomenon can be appreciated in Figure 14. At K’-band, the frequency response compensation is needed to check that it is free of RFI, at least in this measurements.

**Figure 15:**
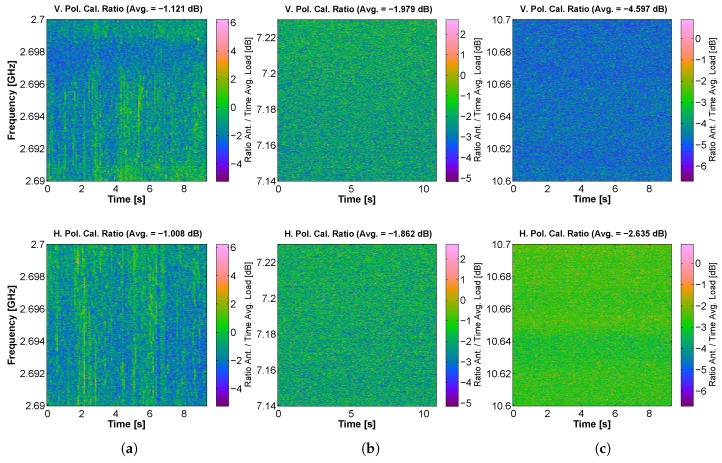

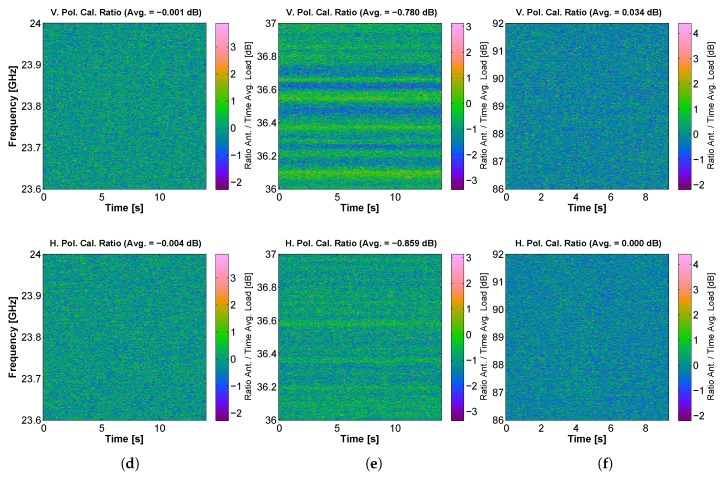
Capture of RFI signals at (**a**) S-; (**b**) C-; (**c**) X-; (**d**) K”-; (**e**) Ka- and (**f**) W-band. All captures are spectrograms with the frequency response compensation procedure already applied. The upper subfigures correspond to V polarization, and the lower ones to H polarization.

The spectrograms corresponding to the S-, C-, X-, K”-, Ka- and W-band at both V and H polarizations, with the frequency response compensation procedure already applied are shown in Figure 15. Regarding the S-band, it can be appreciated that it is even more contaminated than L-band. In this case, the RFI signal is spread in time and frequency for both polarizations. Moreover, the contamination due to the adjacent services, such as WiFi hotspots, is also common at S-band.

Furthermore, the rest of the bands supported by MERITXELL are much cleaner of RFI signals than L- and S-band. The captures at X-band show a uniform RFI contribution in the H polarization over all the frequency band. This kind of RFI signals is commonly caused by the adjacent satellite communication services. Besides, Ka-band shows some continuous wave RFI signals at both polarizations.

In summary, the results show that the lower frequency bands are more contaminated than the higher ones due to the frequency of operation of the wide variety of electronic devices near to L- and S-band. Higher bands seem to be cleaner, not only because of the higher frequency, but also because of the much wider bandwidth, so a narrow band interference occupies a much smaller fraction of the band. However, some RFI signals have also been identified at X- and Ka-band. Eventually, in [22], a similar RFI survey was carried out with similar results.

### 5.4. RFI Example at GPS Bands

**Figure 16:**
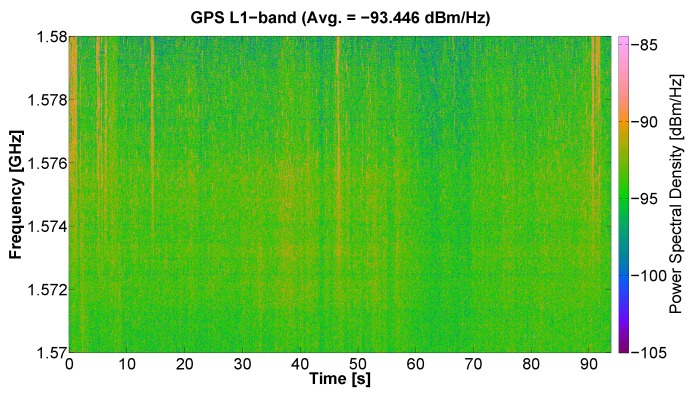
Capture of RFI signals at GPS L1 band using the L-band radiometric antenna.

Since a GNSS-R instrument is installed in MERITXELL, a measurement of the RFI signals present at GPS L1 band was also performed using the radiometric L-band antenna. Figure 16 shows that some pulsed RFI signals are also observed at the GPS L1 band. These RFI signals can still be seen despite the attenuation introduced by the antenna mismatch present when using the radiometric L-band antenna to measure at GPS L1 band. The effect of RFI signals in GPS navigation application is not as critical as the effect on MWR, due to the inherent protection of the GNSS signals. However, for GNSS-R applications, the presence of RFI signals is much critical as compared to navigation applications [37].

## 6. Summary and Conclusions

The present work has shown the hardware and software design of MERITXELL, and the methods and procedures necessary to control, calibrate and perform measurements with it. MERITXELL is a multifrequency instrument that includes a multiband dual-polarization microwave radiometer, a GPS reflectometer, and several optical sensors. The main goal of MERITXELL is twofold. On one hand, it is an outstanding platform to perform detection, characterization, and localization of RFI signals at the most common MWR bands up to 92 GHz. On the other hand, its multisensor architecture enables it to test several data fusion algorithms in post-processing.

## Figures and Tables

**Table 1:** Suitable frequency bands for most prominent MWR applications. Selected frequency bands for each application have been derived from [1,2].

Application	Frequency (GHz)
Clouds water content	21, 37, 90
Ice classification	10, 18, 37
Sea oil spills tracking	6.6, 37
Rain over soil	18, 37, 55, 90, 180
Rain over the ocean	10, 18, 21, 37
Sea ice concentration	18, 37, 90
Sea surface temperature	6.6, 10, 18, 21, 37
Sea surface wind speed	10, 18
Snow coating	6.6, 10, 18, 37, 90
Soil moisture	1.4, 6.6
Atmospheric temperature profiles	21, 37, 55, 90, 180
Atmospheric water vapor	21, 37, 90, 180
Vegetation Water Content	1.4
Land surface temperature	7, 10
Biomass	7, 10, 19

**Table 2:** Main parameters of the eight dual-polarization microwave antennas of MERITXELL. They have been measured at UPC anechoic chamber as mounted in the whole system.

Frequency Band		Beamwidth	$\eta_{\mathrm{MBE}}$	CPI	$\eta_{A}$
L	1.400–1.427 GHz	∼25${}^{\circ }$	95%	35 dB	∼95%
S	2.690–2.700 GHz	∼25${}^{\circ }$	95%	35 dB	∼95%
C	7.140–7.230 GHz	∼25${}^{\circ }$	95%	35 dB	∼95%
X	10.60–10.70 GHz	6${}^{\circ }$	98%	40 dB	∼99%
K	18.60–18.80 GHz	5${}^{\circ }$	98%	40 dB	∼99%
K	23.60–24.00 GHz	4${}^{\circ }$	98%	40 dB	∼99%
Ka	36.00–37.00 GHz	4${}^{\circ }$	98%	40 dB	∼99%
W	86.00–92.00 GHz	3.2${}^{\circ }$	98%	37 dB	∼99%

**Table 3:** Optimal integration time and Allan deviation for each the frequency band and polarization.

	Horizontal Polarization		Vertical Polarization	
Frequency Band	Integration Time (s)	Allan Deviation ($\times 10^{-3}$)	Integration Time (s)	Allan Deviation ($\times 10^{-3}$)
L	193	1.73	142	1.44
S	35	2.77	49	2.30
C	55	2.49	16	3.83
X	89	1.93	34	3.18
K’	29	3.62	13	4.89
K”	55	2.48	77	2.55
Ka	50	2.79	6	6.29
W	13	7.14	17	4.56

## Supplementary Materials

Supplementary File 1

## Abbreviations

The following abbreviations are used in this manuscript:

ADC	Analog-to-Digital Converter
AMSR-E	Advanced Microwave Scanning Radiometer for the Earth Observing System
CIR	Color-Infrared
CPI	Cross-Polarization Isolation
FOW	Field-Of-View
GNSS	Global Navigation Satellite Systems
GNSS-R	Global Navigation Satellite Systems - Reflectometry
GPS	Global Positioning System
GUI	Graphical User Interface
HUTRAD	Helsinki University of Technology RADiometer
I/Q	In-phase and Quadrature
IF	Intermediate Frequency
LAURA	L-band AUtomatic RAdiometer
LHCP	Left-Hand Circularly Polarized
LO	Local Oscillator
MBE	Main Beam Efficiency
MFT	Multiresolution Fourier Transform
MWR	Microwave Radiometry
NIR	Near-Infrared
OMT	Orthomode Transducer
PAU-SA	Passive Advanced Unit - Synthetic Aperture
PID	Proportional Integral Derivative
PLC	Programmable Logic Controller
PSR	Polarimetric Scanning Radiometer
R&S	Rohde & Schwarz
RBW	Resolution Bandwidth
RFI	Radio-Frequency Interference
RGB	Red-Green-Blue
RMS	Root Mean Square
RSLab	Remote Sensing Laboratory
SA	Spectrum Analyzer
SMOS	Soil Moisture and Ocean Salinity
SPDT	Single-Pole Dual-Through
SSMI/S	Special Sensor Microwave Imager/Sounder
TIR	Thermal Infrared
TPR	Total-Power Radiometer
UPC	Polytechnic University of Catalonia - BarcelonaTech
VBW	Video Bandwidth
VIS/IR	Visible/Infrared
